# Resting Oxygen Consumption Estimates in Scleroderma Can Lead to Underestimation of Cardiac Output

**DOI:** 10.1002/pul2.70280

**Published:** 2026-03-10

**Authors:** Oscar Cullen, Laura Ross, Jessica L. Fairley, Luke W. Spencer, Amy M. Mitchell, Stephanie J. Rowe, Kristel Janssens, Youri Bekhuis, Stephen J. Foulkes, Paolo D'Ambrosio, Margarita Calvo‐Lopez, Kaitlin Newcomb, Andre La Gerche, Andrew T. Burns

**Affiliations:** ^1^ HEART Lab, St Vincent's Institute of Medical Research Fitzroy Australia; ^2^ Department of Rheumatology St Vincent's Hospital Melbourne Fitzroy Australia; ^3^ Department of Medicine University of Melbourne Parkville Australia; ^4^ Department of Cardiology St Vincent's Hospital Melbourne Fitzroy Australia; ^5^ Exercise and Nutrition Research Program The Mary MacKillop Institute for Health Research, ACU Melbourne Australia; ^6^ Department of Cardiovascular Diseases KU Leuven Leuven Belgium; ^7^ Faculty of Nursing University of Alberta Edmonton Canada; ^8^ Department of Cardiology, John Hunter Hospital New Lambton Heights Australia; ^9^ Department of Cardiology Hospital Puerta de Hierro Majadahonda Spain

**Keywords:** indirect fick, oxygen consumption, pulmonary arterial hypertension, scleroderma

## Abstract

Accurate resting oxygen consumption (rVO_2_) quantification is critical for Fick‐derived cardiac output calculations. Yet, clinical practice predominantly uses empirical estimations, which can be inaccurate. We evaluated the established rVO₂ prediction equations against direct metabolic cart measurements in systemic sclerosis patients, revealing significant discordances with potential diagnostic implications.

## 1 Introduction

Systemic sclerosis (SSc) is a rare connective tissue disease that has a global prevalence of 23 per 100,000 and disproportionately affects women [1]. Complex cardiopulmonary disease can occur in SSc, including pulmonary arterial hypertension (PAH), which is diagnosed in up to 15% of all SSc patients and is a leading cause of death [2]. PAH results from pathological remodelling of the pulmonary vasculature that can result in increasing right ventricular afterload, potentially leading to progressive right heart failure. PAH is classified as a mean pulmonary arterial pressure of ≥ 20 mmHg with pulmonary artery wedge pressure (PAWP) < 15 mmHg and pulmonary vascular resistance (PVR) of > 2 Wood Units at right heart catheterisation (RHC) [3]. With only ~50% of SSc patients surviving after 5 years of PAH diagnosis, it is imperative that SSc patients are routinely screened for PAH and further investigated with RHC to confirm the diagnosis and commencement of pulmonary vasodilator therapy [4].

During RHC, there are two methods to measure cardiac output (CO): (1) indicator dilution methods such as thermodilution and (2) the Fick method [CO = oxygen uptake (VO_2_)/arteriovenous oxygen difference (avO_2_‐diff)]. VO_2_ can be obtained through breath‐by‐breath mixed gas analysis from a metabolic cart, known as the direct Fick method or estimated using a formula. (3) Accurate assessment of resting oxygen consumption (rVO_2_) is crucial for the accurate quantification of CO and PVR. However, direct measurement of rVO_2_ requires specialised equipment and is frequently simplified using the ‘indirect’ Fick method in which rVO_2_ is estimated using predictive nomograms that integrate anthropometric parameters including body surface area (BSA), age, sex, and heart rate. These equations have drawn some criticism as they have been derived from studies in paediatric populations or ostensibly healthy adult cohorts that have relatively stable metabolism [5, 6, 7, 8, 9, 10]. These populations do not accurately reflect the complex, multimorbid patients that typically present at a cardiac catheterisation laboratory for RHC investigation. Hence, there is concern that the application of rVO_2_ estimations may lead to inaccurate CO measurements in at‐risk populations.

To our knowledge, indirect Fick methods have not been validated in SSc. This gap in the literature could lead to population‐specific miscalculations, misdiagnosis, and inadequate patient management. Therefore, we aimed to evaluate the relationship between these estimated rVO_2_ equations and directly measured values in a SSc population.

## 2 Methods

We performed a retrospective data analysis from a pooled dataset of two studies (Breathlessness in systemic sclerosis—quantifying the contribution of cardiac fibrosis; Defining the burden of arrhythmias in systemic sclerosis: a cohort control study) on 76 patients with a confirmed ACR/EULAR SSc diagnosis that completed testing at one of two sites: (1) St Vincent's Hospital Melbourne, Australia; (2) Baker Institute, Melbourne, Australia. The St Vincent's Hospital Human Research Ethics Committee approved both studies (local HREC numbers: 181‐18 and 062‐22), which were conducted in accordance with the Declaration of Helsinki, and all participants provided informed consent.

Directly measured rVO_2_ data was collected whilst seated on an upright bike and averaged over a 2‐min rest period from breath‐by‐breath gas analysis with a calibrated metabolic cart (Vyntus CPX, Jaeger Medical GmbH, Hoechberg Germany). Estimated rVO2 values were derived from three empirical equations by:

1. La Farge & Miettinen [11] [VO_2_ (mL/min) = 138.1 − (X × log age) + (0.378 × HR) × BSA (m^2^)] (Men: X = 11.49; Women: X = 17.04);
2. Dehmer et al. [12] [VO_2_ (mL/min) = 125 (mL/min/m^2^) × BSA (m^2^)];
3. Bergstra et al. [13] [VO_2_ (mL/min) = 157.3 × BSA + X − (10.5 × log age) + 4.8] (Men: X = 10; Women = 0).

A paired samples t‐test was performed to test differences between measured rVO_2_ values and estimates, and an independent samples *t*‐test was utilised to assess the sex differences in the SSc cohort. Scatterplots with Pearson's *r* correlation were used to measure the strength of the association, and Bland–Altman plots were used to assess the agreement between the values. All statistical analyses were conducted with an open‐source statistics platform JAMOVI v2.5 (The Jamovi Project, Sydney, Australia). The composite six‐panel figure was created using R v4.03 (R Foundation for Statistical Computing, Vienna, Austria). For all tests, a *p*‐value of < 0.05 was deemed statistically significant and < 0.001 was deemed strongly statistically significant.

## 3 Results

The mean age for the SSc cohort was 56 ± 11 years, with males (*n* = 15) slightly older (59 ± 9 years) than females (*n* = 61) (55 ± 11 years; *p* = 0.142). The majority were Caucasian (*n* = 72), with 41 participants having limited cutaneous SSc, while 35 participants had diffuse cutaneous SSc. The mean disease duration was 11 ± 9 years, with the largest comorbidity being dyslipidaemia with 26%, followed by interstitial lung disease (ILD) in 14%, and notably, PAH was present in 5% of the cohort.

For the entire SSc cohort, measured rVO_2_ was 309.9 ± 75.7 mL/min, whereas estimated rVO_2_ was 166.5 ± 11 mL/min (*p* ≤0.001) calculated with La Farge & Miettinen equation; 225.6 ± 21.1 mL/min (*p* ≤ 0.001) with the Dehmer et al. equation; and 272.4 ± 28 mL/min (*p* ≤ 0.001) using the Bergstra et al. equation.

When separated by sex, females had a lower measured rVO_2_ compared to males [(females: 228.8 ± 65.7 mL/min) (males: 394.6 ± 50.3 mL/min; *p* ≤ 0.001)]. The estimated measures for the females were also lower for each equation comparatively to males; La Farge & Miettinen: 164.4 ± 10.2 versus 175.1 ± 10.3 mL/min, Dehmer et al.: 222.0 ± 21.4 versus 240.3 ± 15.3 mL/min, Bergstra et al.: 266.0 ± 26.9 versus 298.7 ± 15.3 mL/min, all *p* < 0.01. All estimated equations had smaller mean differences between rVO_2_ and estimated VO_2_ for females compared to males, however, similar limits of agreement (LOA). Bergstra et al. was the closest [females: 22.5 mL/min (LOA: −86.8 to 131.8 mL/min); males: 96.0 mL/min (LOA: −2.5 to 194.4 mL/min)] and La Farge & Miettinen was the farthest [females: 124.6 mL/min (LOA: 0.3–248.8 mL/min); males: 219.6 mL/min (LOA: 128.0–311.1 mL/min)] for both groups.

**Figure 1:**
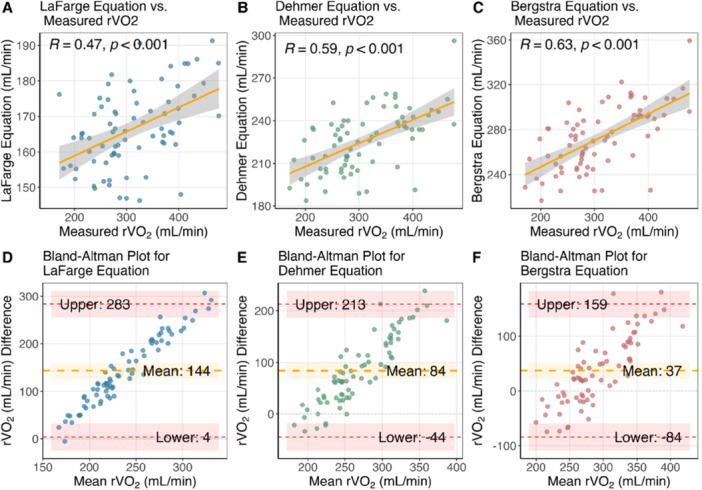
Top row showcases three scatterplots comparing directly measured rVO₂ with estimated rVO_2_ by the LaFarge (A), Dehmer (B) and Bergstra (C) equations. The bottom row shows the corresponding Bland–Altman plots (LaFarge, Dehmer and Bergstra equations as panels D, E and F respectively) with mean differences (middle yellow dashed line) and limits of agreement (the uppermost and lowermost red dashed lines) with 95% confidence intervals, which are illustrated as the highlighted area.

**Figure 2:**
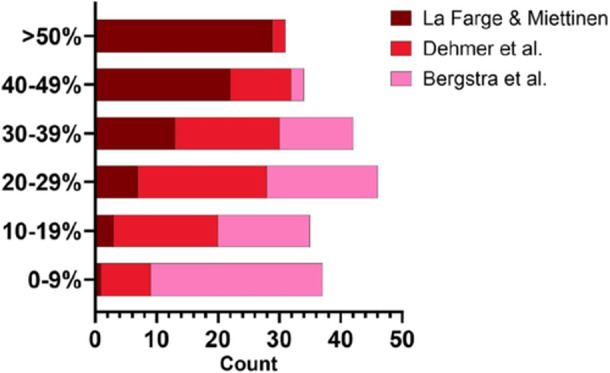
Bar chart that illustrates the percentage differences between the estimated equations and measured rVO_2_ that is categorised based on the distribution of the difference.

Scatterplots and Bland–Altman plots comparing each equation to directly measured values are illustrated in Figure 1. Correlation analysis revealed Bergstra et al.'s equation to have the strongest agreement, Dehmer et al. demonstrated a moderate agreement, while La Farge & Miettinen had the weakest. The bar chart that is illustrated in Figure 2, showed Bergstra et al. to also be the one with the smallest distribution of percentage differences. Although all equations provided large differences from measured values, with the majority of cases above a 20% difference.

## 4 Discussion

Our findings demonstrate systematic underestimation of rVO_2_ by each of the empiric formulae compared to directly measured values. Our findings in this large SSc cohort confirm the need for accurately acquiring direct rVO_2_ instead of relying on inaccurate rVO_2_ estimates. This is consistent with a growing body of evidence highlighting how rVO_2_ estimates do not accurately quantify directly measured rVO_2_ [5, 6, 7, 8, 9, 10, 14]. Interestingly, while we showed estimations tend to underestimate rVO_2_ in SSc, Grafton et al. showed that rVO_2_ formulae tended to overestimate direct rVO_2_ in patients with heart failure. This discrepancy could reflect potential disease‐specific differences in resting metabolic state, but could also arise from differences in study methodology and sample characteristics. Regardless, the poor performance of rVO_2_ estimates in both studies highlights the imperative of direct rVO_2_ measurements. To our knowledge, this is the first study to show these equations to be inaccurate in SSc. Previous studies have shown that while peak exercise VO_2_ in patients with SSc is reduced relative to controls, there is no recorded discernible difference in rVO_2_. However, ours is the first study to evaluate whether rVO_2_ estimations can be accurately applied on an individual basis to patients with SSc. This has potentially large clinical implications as the usage of these equations during RHC investigations could underestimate the patient's true rVO_2_, suggesting a higher PVR and subsequently misclassifying SSc patients with PAH.

Consistent with prior studies, we observed a significant sex difference that resulted in greater inaccuracies in males, particularly when using the Dehmer et al. and Bergstra et al. equations [5, 7]. This may lead to sex‐specific differences in the diagnostic utility of using the Fick method of calculating CO. This may be due to limitations of the formulas used. Equally, it may be due to sex‐specific differences in SSc patients, given that men have a tendency for a more rapidly progressive disease state [15, 16]. Hence, in this context, there may well be sex‐specific differences in pulmonary haemodynamics that could be an interesting area for future research.

To combat the practicality issue of obtaining directly measured rVO_2_, there has been growing interest in easy‐to‐use portable equipment [17, 18]. If these devices are proven to be valid and reproducible, this could be an easily implementable solution [17, 18]. Thermodilution remains a valid method for measuring CO; however, it may be less accurate in the context of severe tricuspid regurgitation or low CO state and intracardiac shunts [19, 20].

There are some limitations to this study. This includes the retrospective design, and the lack of invasive blood gas assessments that prevented the calculation of PVR. However, given our study showed the error in rVO_2_ was as high as 283 mL/min for some individuals, our findings demonstrate the substantial potential for PAH misclassification with rVO_2_ equations. As this was a secondary analysis, methodological constraints meant that rVO_2_ was assessed in the upright rather than supine position. However, we believe it still accurately reflects the inter‐individual variability of rVO_2_.

## 5 Conclusion

Empiric equations systemically underestimate rVO_2_ and should be avoided as much as practicable. These errors may impact treatment decisions in the context of PAH diagnosis. When direct measurement is unavailable, thermodilution should be the preferred method.

## Author Contributions

**Oscar Cullen:** investigation (supporting), project administration (supporting), writing – original draft (lead), formal analysis (lead), writing – review and editing (equal). **Laura Ross:** investigation (lead), methodology (lead), project administration (lead), formal analysis (supporting), writing – review and editing (equal). **Jessica L. Fairley:** investigation (lead), methodology (lead), project administration (lead), formal analysis (supporting), writing – review and editing (equal). **Luke W. Spencer:** visualisation, validation, writing – review and editing (equal). **Amy M. Mitchell:** writing – review and editing (equal). **Stephanie J. Rowe:** writing – review and editing (equal). **Kristel Janssens:** writing – review and editing (equal). **Youri Bekhuis:** writing – review and editing (equal). **Stephen Foulkes:** writing – review and editing (equal). **Paolo D'Ambrosio:** writing – review and editing (equal). **Margarita Calvo‐Lopez:** writing – review and editing (equal). **Kaitlin Newcomb:** writing – review and editing (equal). **Andre La Gerche:** conceptualisation (supporting), resources (lead), methodology (supporting), supervision (lead), formal analysis (supporting), writing – review and editing (equal). **Andrew T. Burns:** conceptualisation (lead), supervision (supporting), writing – original draft (supporting), formal analysis (supporting), writing – review and editing (equal).

## Ethics Statement

The St Vincent's Hospital Human Research Ethics Committee approved both studies (local HREC numbers: 181‐18 and 062‐22), which were conducted in accordance with the Declaration of Helsinki, and all participants provided informed consent.

## Conflicts of Interest

J.L.F. ‐ Speakers Bureau honoraria, 2023 (Boehringer‐Ingelheim), conference support 2021 (Pfizer) (2021).

## Guarantor

Not applicable.

## References

[1] L. M. Calderon and J. E. Pope , “Scleroderma Epidemiology Update,” Current Opinion in Rheumatology 33, no. 2 (2021): 122–127. 33481429 10.1097/BOR.0000000000000785

[2] J. L. Fairley , L. Ross , E. Paratz , et al., “Pathological Contributors to Organ Damage and Mortality in Systemic Sclerosis: A Nationwide Matched Case‐Control Study,” Seminars in Arthritis and Rheumatism 73 (2025): 152739. 40344933 10.1016/j.semarthrit.2025.152739

[3] M. Humbert , G. Kovacs , M. M. Hoeper , et al., “2022 ESC/ERS Guidelines for the Diagnosis and Treatment of Pulmonary Hypertension,” European Respiratory Journal 61, no. 1 (2023): 2200879. 36028254 10.1183/13993003.00879-2022

[4] K. Morrisroe , W. Stevens , M. Huq , et al., “Survival and Quality of Life in Incident Systemic Sclerosis‐Related Pulmonary Arterial Hypertension,” Arthritis Research & Therapy 19, no. 1 (2017): 122. 28576149 10.1186/s13075-017-1341-x PMC5457656

[5] P. J. Chase , P. G. Davis , L. Wideman , J. W. Starnes , M. R. Schulz , and D. R. Bensimhon , “Comparison of Estimations Versus Measured Oxygen Consumption at Rest in Patients With Heart Failure and Reduced Ejection Fraction Who Underwent Right‐Sided Heart Catheterization,” American Journal of Cardiology 116, no. 11 (2015): 1724–1730. 26443561 10.1016/j.amjcard.2015.08.051

[6] P. Agasthi , W. Miranda , and A. Egbe , “Discordance Between Measured vs Calculated Oxygen Consumption in Adults With Congenital Heart Disease: Limitations and Clinical Implications,” Journal of Invasive Cardiology 33, no. 2 (2021): E100–E107. 33531441 10.25270/jic/20.00387

[7] N. Narang , M. O. Gore , P. G. Snell , et al., “Accuracy of Estimating Resting Oxygen Uptake and Implications for Hemodynamic Assessment,” American Journal of Cardiology 109, no. 4 (2012): 594–598. 22100029 10.1016/j.amjcard.2011.10.010 PMC13150820

[8] N. Narang , J. T. Thibodeau , B. D. Levine , et al., “Inaccuracy of Estimated Resting Oxygen Uptake in the Clinical Setting,” Circulation 129, no. 2 (2014): 203–210. 24077170 10.1161/CIRCULATIONAHA.113.003334

[9] U. Fakler , C. Pauli , M. Hennig , W. Sebening , and J. Hess , “Assumed Oxygen Consumption Frequently Results in Large Errors in the Determination of Cardiac Output,” Journal of Thoracic and Cardiovascular Surgery 130, no. 2 (2005): 272–276. 16077386 10.1016/j.jtcvs.2005.02.048

[10] A. H. Kendrick , J. West , M. Papouchado , and A. Rozkovec , “Direct Fick Cardiac Output: Are Assumed Values of Oxygen Consumption Acceptable?,” European Heart Journal 9, no. 3 (1988): 337–342. 3383873 10.1093/oxfordjournals.eurheartj.a062505

[11] C. G. LaFarge and O. S. Miettinen , “The Estimation of Oxygen Consumption,” Cardiovascular Research 4, no. 1 (1970): 23–30. 5416840 10.1093/cvr/4.1.23

[12] G. J. Dehmer , B. G. Firth , and L. D. Hillis , “Oxygen Consumption in Adult Patients During Cardiac Catheterization,” Clinical Cardiology 5, no. 8 (1982): 436–440. 7127921 10.1002/clc.4960050803

[13] A. Bergstra , R. B. Van Dijk , H. L. Hillege , K. I. Lie , and G. A. Mook , “Assumed Oxygen Consumption Based on Calculation From Dye Dilution Cardiac Output: An Improved Formula,” European Heart Journal 16, no. 5 (1995): 698–703. 7588904 10.1093/oxfordjournals.eurheartj.a060976

[14] G. Grafton , T. M. Cascino , D. Perry , C. Ashur , and T. M. Koelling , “Resting Oxygen Consumption and Heart Failure: Importance of Measurement for Determination of Cardiac Output With the Use of the Fick Principle,” Journal of Cardiac Failure 26, no. 8 (2020): 664–672. 30753933 10.1016/j.cardfail.2019.02.004 PMC6689258

[15] P. E. Carreira , L. Carmona , B. E. Joven , et al., “Gender Differences in Early Systemic Sclerosis Patients: A Report From the EULAR Scleroderma Trials and Research Group (EUSTAR) Database,” supplement, Clinical and Experimental Rheumatology 36, no. Suppl 113 (2018): S68–S75. 30277860

[16] M. Elhai , J. Avouac , U. A. Walker , et al., “A Gender Gap in Primary and Secondary Heart Dysfunctions in Systemic Sclerosis: A EUSTAR Prospective Study,” Annals of the Rheumatic Diseases 75, no. 1 (2016): 163–169. 25342760 10.1136/annrheumdis-2014-206386

[17] S. E. Crouter , S. R. LaMunion , P. R. Hibbing , A. S. Kaplan , and D. R. Bassett , “Accuracy of the Cosmed K5 Portable Calorimeter,” PLoS One 14, no. 12 (2019): e0226290. 31841537 10.1371/journal.pone.0226290 PMC6913985

[18] J. S. Thiessen , N. A. Guluzade , R. Faricier , and D. A. Keir , “(Re) Assessment of the COSMED Quark CPET and VO2Master Pro Systems for Measuring Pulmonary Gas Exchange and Ventilation,” Scandinavian Journal of Medicine & Science in Sports 35, no. 2 (2025): e70019. 39888076 10.1111/sms.70019 PMC11780302

[19] G. W. Hamilton , L. Fletcher , W. Harley , et al., “Agreement Between Direct Fick and Bolus Thermodilution Measurements of Cardiac Output During Right Heart Catheterisation and the Impact on Haemodynamic Classifications,” supplement, European Heart Journal 44, no. Suppl_2 (2023): ehad655.

[20] N. Narang , J. T. Thibodeau , W. F. Parker , et al., “Comparison of Accuracy of Estimation of Cardiac Output by Thermodilution Versus the Fick Method Using Measured Oxygen Uptake,” American Journal of Cardiology 176 (2022): 58–65. 35613956 10.1016/j.amjcard.2022.04.026 PMC9648100

